# Cellular heterogeneity in red and melanized focal muscle changes in farmed Atlantic salmon (*Salmo salar*) visualized by spatial transcriptomics

**DOI:** 10.1007/s00441-023-03850-x

**Published:** 2023-12-13

**Authors:** H. Bjørgen, S. Malik, E. Rimstad, M. Vaadal, I. B. Nyman, E. O. Koppang, T. Tengs

**Affiliations:** 1 Unit of Anatomy, Faculty of Veterinary Medicine, Norwegian University of Life Sciences, 1433 Ås, Norway; 2 Department of Breeding and Genetics, Nofima, 1433 Ås, Norway; 3 Unit of Virology, Faculty of Veterinary Medicine, Norwegian University of Life Sciences, 1433 Ås, Norway

**Keywords:** Atlantic salmon, Melanization, Spatial transcriptomics, PRV-1, Hypoxia

## Abstract

Spatial transcriptomics is a technique that provides insight into gene expression profiles in tissue sections while retaining structural information. We have employed this method to study the pathological conditions related to red and melanized focal changes in farmed Atlantic salmon (*Salmo salar*). Our findings support a model where similar molecular mechanisms are involved in both red and melanized filet discolorations and genes associated with several relevant pathways show distinct expression patterns in both sample types. Interestingly, there appears to be significant cellular heterogeneity in the foci investigated when looking at gene expression patterns. Some of the genes that show differential spatial expression are involved in cellular processes such as hypoxia and immune responses, providing new insight into the nature of muscle melanization in Atlantic salmon.

**Supplementary Information:**

The online version contains supplementary material available at 10.1007/s00441-023-03850-x.

## Introduction

**Fig. 1:**
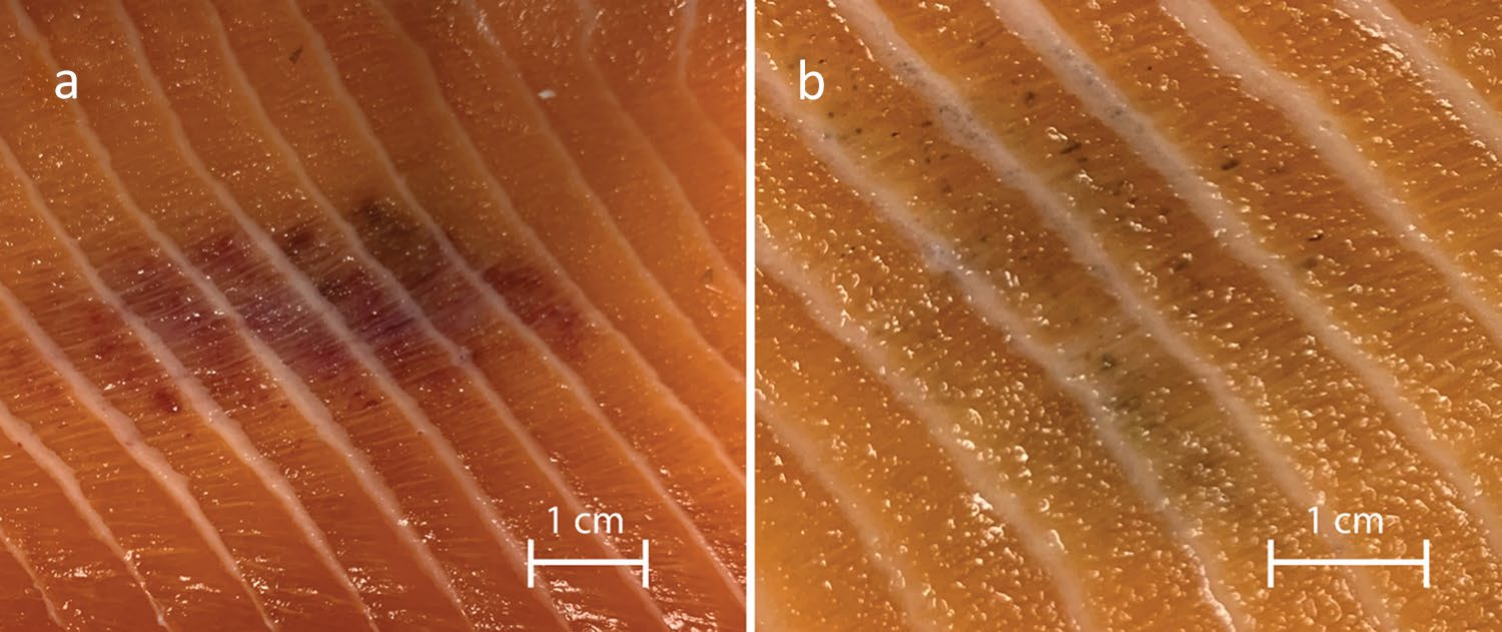
Red focal changes (RFCs) (**a**) and melanized focal changes (MFCs) (**b**) in Atlantic salmon fillet

Melanization of the white muscle (i.e., the *filet*) is a significant quality problem in farmed Atlantic salmon (*Salmo salar*) (Larsen et al. 2012). The melanized changes appear as focal spots, typically a few centimeters in diameter, predominantly in the cranio-ventral region of the abdominal musculature (Bjorgen et al. 2019). The prevalence of melanized focal changes (MFCs) varies across fish populations and farms, with a reported average prevalence of 20% at slaughter (Morkore and Heia 2012). Histologically, MFCs, at the time of slaughter, were first characterized as chronic inflammatory changes infiltrated with melano-macrophages, which are pigment-producing leukocytes causing the localized discoloration (Koppang et al. 2005; Larsen et al. 2012). However, later studies demonstrated that these macroscopic focal changes could be divided into nine categories with differing histological characteristics (Bjorgen et al. 2019). The changes appear to follow a progression from an acute stage characterized by hemorrhages and necrosis, visually appearing as red focal changes (RFCs), to MFCs showing a gradual progression of the abundance of melano-macrophages in affected musculature in combination with different stages of inflammation that either resolves or enters a chronic, granulomatous stage (Bjorgen et al. 2015) (Fig. 1). Despite numerous suggested causes (Bjorgen et al. 2020; Brimsholm et al. 2023; Jimenez-Guerrero et al. 2023), the underlying etiology of RFC and its subsequent progression to MFC remains elusive.

The discoloration in the dark foci is due to the presence of melano-macrophages (Sichel et al. 1997), which appear in connection with different diseases in fish (Agius and Roberts 2003). Such cells are common in visceral immune organs of fish and are thought to be melanin-producing macrophages (Bjorgen and Koppang 2021). Gene expression analyses have been conducted on both MFCs and RFCs, and increased expression of genes involved in the melanogenesis pathway has been observed in MFCs, indicating de novo synthesis of melanin within these changes (Larsen et al. 2013). In RFCs, innate immune genes are upregulated, while genes associated with adaptive immunity show increased expression in MFCs (Bjorgen et al. 2020). Macrophages are the main immune cells in granulomatous inflammatory changes (Malik et al. 2021a), and the upregulation of MHC class II observed is likely related to the presence of such cells (Larsen et al. 2013; Bjorgen et al. 2020). Additionally, upregulation of CD4 transcripts suggests interactions between CD4^+^ T cells and MHC class II^+^ cells. Transcriptome profiling of MFCs has revealed similar findings, along with robust B cell responses including significant induction of immunoglobulins (Krasnov et al. 2016; Jimenez-Guerrero et al. 2023). Based on these transcriptional studies, an infectious cause, potentially bacterial or viral in nature, has been suspected. Detection of prokaryotic rRNA, viral RNA and proteins from Piscine orthoreovirus 1 (PRV-1) has been reported within the changes, although causation for the initiation of the pathological changes has not been confirmed (Bjorgen et al. 2015; Krasnov et al. 2016). Nevertheless, the presence of PRV-1 seems to be essential for granulomatous development as virus may not only be present within granulomas, but also replicate in situ (Bjorgen et al. 2020; Malik et al. 2021a, b).

Conventional gene expression analyses have uncovered the involvement of different immune genes in these changes. Recent advancements in spatially resolved transcriptomics allow precise mRNA expression profiling within tissue sections, making it particularly suitable for pathological conditions characterized by distinct tissue changes. The objective of this study was to use spatial transcriptomics to investigate gene expression patterns in RFCs and MFCs of farmed Atlantic salmon.

## Materials and methods

### Sample preparation

Samples were collected from farmed Atlantic salmon at slaughter weight (app. 5 kg). Based on visual inspection of filets, four individuals were selected for RFC and MFC sampling (two biological replicates per sample type). Muscle samples measuring 5 mm^3^ were collected on site from the center of the affected tissue regions and placed in a drop of Tissue-TEK O.C.T. Compound (Sakura Finetek, Torrance, CA, USA) on a metal plate on dry ice. The embedded tissue samples were held on dry ice until they were completely frozen and subsequently stored at − 80 °C until sectioning.

Optimization of tissue permeabilization was performed on 10-μm-thick cryosections using Visium Spatial Tissue Optimization Reagents Kit according to the protocol provided (10 × Genomics, Pleasanton, CA, USA), which established the optimal permeabilization time to be 15 min. Samples were mounted onto a Gene Expression slide (10 × Genomics) and stored at − 80 °C until hematoxylin and eosin (H&E) staining. Tissue staining and library preparation was performed according to the manufacturer’s manual. Fifty base pair paired-end libraries were sequenced using the NovaSeq 6000 system with an SP flow cell (Illumina, San Diego, CA, USA), at the Norwegian sequencing center (Oslo, Norway).

### Histological investigation—hematoxylin and eosin stain

Cryosections (4 μm) were prepared from the embedded tissue samples. After air drying for 60 min, the sections were fixed in 4% buffered formalin for 5 min. Following a brief rinse in water, the sections were immersed in Mayer’s hematoxylin for 5 min. After another 5 min water rinse, the sections were stained with eosin for 5 s before being transferred to xylene. The sections underwent ten washes in 70% ethanol, followed by a 30 s immersion in 95% ethanol, and finally left for 1 min in 100% ethanol before mounting.

### Read mapping, cell population identification, and PCR

Reads were aligned to the Atlantic salmon genome (version Ssal_v3.1, INSDC Assembly GCA_905237065.2) (Lien et al. 2016) using the software Space Ranger (version 1.3.1; 10 × Genomics) with default settings. High-resolution JPG images of the tissue sections were generated using a Leica Aperio CS2 Slide Scanner (Leica Biosystems, Deer Park, IL, USA). Downstream analyses were done using the R package Seurat (version 4.3.0) (Hao et al. 2021). Data were transformed using sctransform (Hafemeister and Satija 2019), and cell populations were identified using the shared nearest neighbor (SNN) modularity clustering algorithm (Waltman and van Eck 2013) (https://satijalab.org/seurat/articles/spatial_vignette.html). Data from biological duplicates were combined using Seurat’s “merge” function.

The corresponding Atlantic salmon transcriptome was downloaded and associated with Gene Ontology (GO) terms using the software InterProScan (version 5.51–85.0) (Jones et al. 2014). Gene lists were analyzed for enrichment of GO terms using the R package GOstats (version 2.62.0) (Falcon and Gentleman 2007). For the RFC samples, the GOstats gene universe was defined as all genes showing some level of expression in at least one RFC cell population, and the same approach was used when defining the MFC gene universe.

Additionally, subsets of genes were annotated using the KofamKOALA software (Aramaki et al. 2020) with default settings. KEGG Orthologs (KOs) were extracted and used as input in KEGG Mapper Reconstruct analyses (KEGG Pathway Database Release 105.0) (Kanehisa and Sato 2020).

Real-time PCR was used for detection and quantification of PRV-1 in the samples. Briefly, a small cube (approx. 3 mm^3^) of tissue was dissected from the O.C.T. blocks and RNA was extracted using the RNeasy Fibrous Tissue Mini Kit (QIAGEN, Hilden, Germany). PCR was performed using the Brilliant III Ultra-Fast QRT-PCR Master Mix (Agilent Technologies, Santa Clara, CA, USA) and a previously described protocol (Wessel et al. 2015).

## Results

### Histological investigation—hematoxylin and eosin stain

**Fig. 2:**
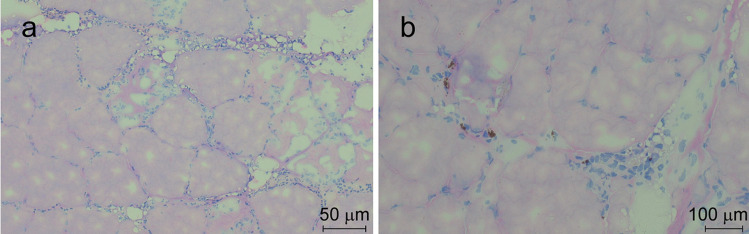
Hematoxylin and eosin stain of **a** RFC and **b** MFC

Sections of RFCs exhibited hemorrhage in the endomysial space, as depicted in Fig. 2a. Degenerated myocytes and infiltrates of immune cells were scattered throughout, while no melano-macrophages were detected. Conversely, in the MFCs, there were noticeable infiltrates of immune cells, including melano-macrophages, as shown in Fig. 2b. Additionally, mild fibrotic changes were observed.

### Spatial transcriptomics

**Fig. 3:**
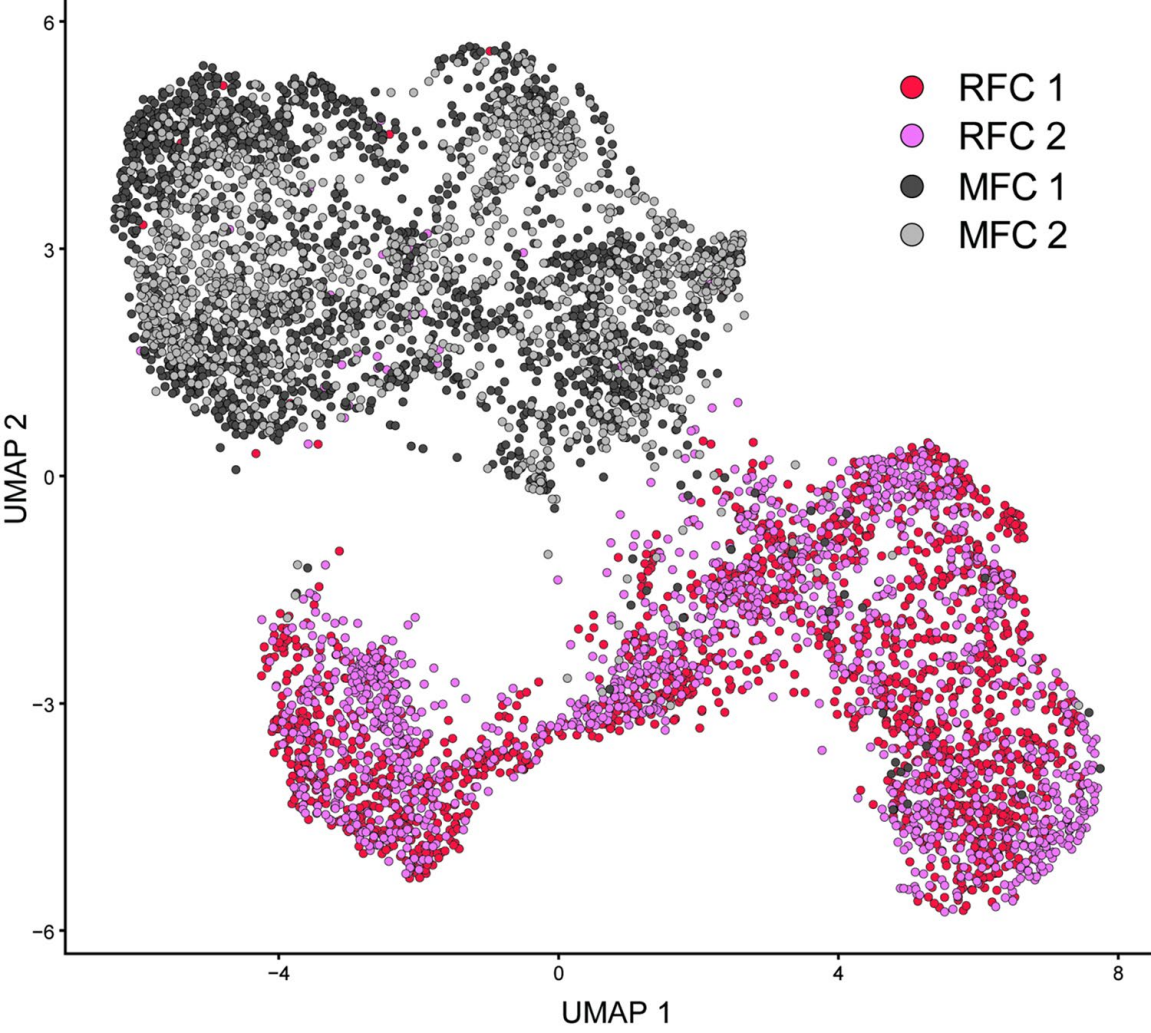
UMAP clustering of all spots with different spatial barcodes on the spatial gene expression slide, based on complete gene expression profiles

**Fig. 4:**
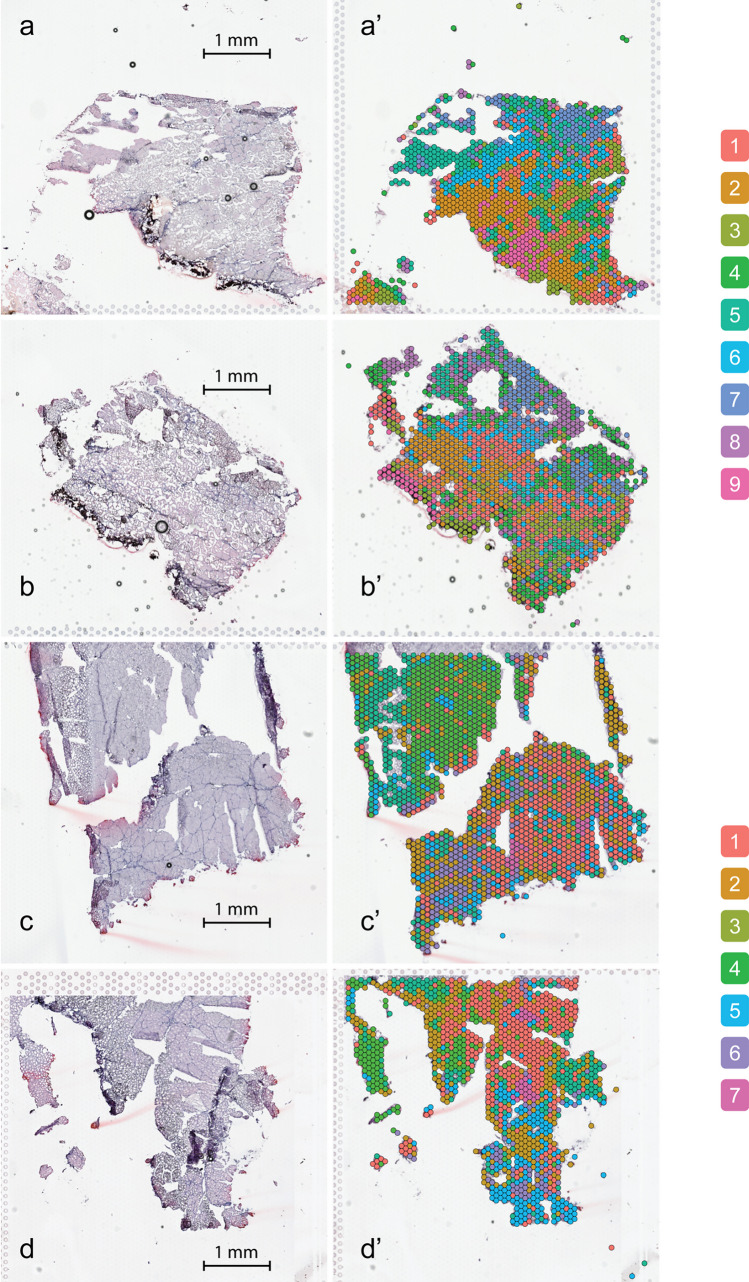
Spatially resolved cell populations as defined by SNN clustering for RFC samples 1 and 2 and MFC samples 1 and 2 (**a**, **b**, **c**, and **d**) and corresponding histology images (a’, b’, c’, and d’)

**Fig. 5:**
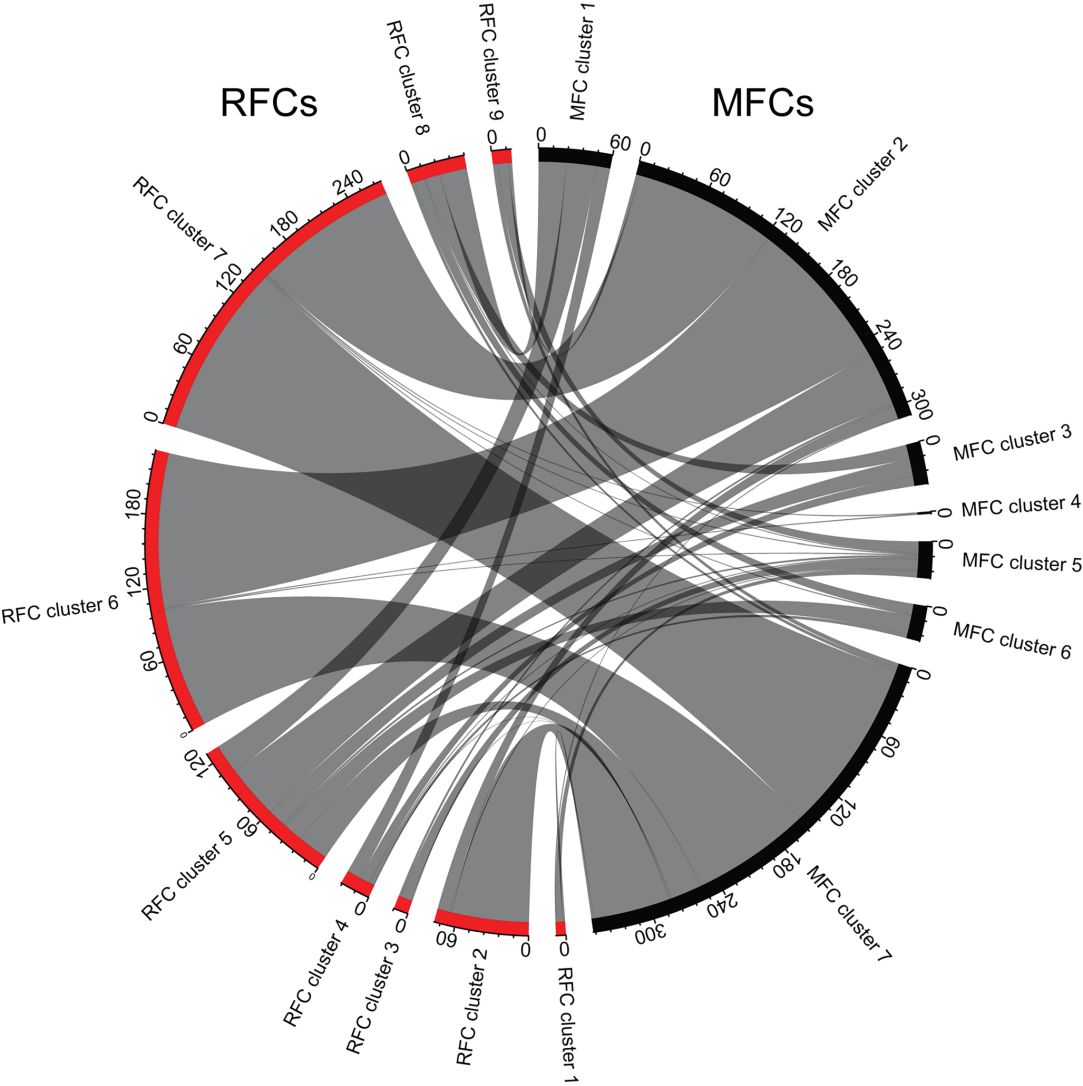
Chord diagram showing the extent of overlap in genes defining the different cell populations (RFCs versus MFCs; up- and downregulated genes combined)

The number of reads from the four samples varied from 107,930,428 to 148,919,112, with mapping efficiency ranging from 91.70 to 94.50% (Table 1). Raw reads were submitted to NCBI’s Sequence Read Archive (SRA) database as BioProject PRJNA983972. Dimensional reduction using uniform manifold approximation and projection (UMAP) of the gene expression profiles for all the spots from the four sections showed clustering of spots from the two sample types, indicating a distinct set of gene expression patterns in RFCs versus MFCs (Fig. 3). Using SNN clustering of individual sections, similar numbers of cell populations were identified in RFCs versus MFCs, and based on these observations, the biological duplicates were merged, and cell populations identified for the two combined datasets. For the RFCs, a total of nine populations were then defined using SNN clustering, whereas the analysis of the MFC samples indicated seven distinct cell populations (Fig. 4). The genes defining the cell populations overlapped significantly for several of the clusters (Fig. 5). The largest overlap was seen between RFC cluster 7 and MFC cluster 6, with a total of 184 overlapping genes (Fig. 5).

**Table 1:** Mapping statistics for the spatial transcriptomics analysis

**Sample**	**Number of spots under tissue**	**Median genes per spot**	**Mean reads per spot**	**Number of reads**	**Reads mapped to genome**
RFC 1	1664	854	64,862	107,930,428	94.50%
RFC 2	1460	496	86,228	125,892,600	93.20%
MFC 1	1801	501	82,687	148,919,112	91.70%
MFC 2	1320	419	89,834	118,580,558	92.10%

Functional annotation of the genes defining the different cell populations mostly showed enrichment of higher order GO terms (Table 2, Supplementary Table 1). Looking at “Biological process” and “Molecular function,” the most significantly enriched terms were “Translation” and “Structural constituent of ribosome,” respectively, in RFC cluster 6 (Table 2). All the genes listed as members of either RFC or MFC cell populations were also associated with KOs (adjusted *p* value < 0.05; Supplementary Table 2), to be used in KEGG Mapper Reconstruct analyses. The 500 genes with the lowest adjusted *p* values (cutoff approximately 3.95 × 10^−48^; Supplementary Table 2) were used as input. The highest number of “Immune system” orthologs was found for “Antigen processing and presentation” (4 KOs; Table 3; Supplementary Fig. 1), with the highest number of differentially expressed genes (DEGs) listed for MFC cluster 7 and RFC cluster 7, with five immune genes showing a significantly higher level of expression in these cell populations when contrasted with the rest of the MFC sections. In the category “Environmental Information Processing,” the highest number of orthologs was found for the HIF-1 signaling pathway (7 KOs; 15 genes; Table 3; Supplementary Fig. 2) with more evenly distributed numbers of DEGs across cell populations (Table 3).

**Table 2:** Top scoring GO term for the different cell populations (“Biological process” and “Molecular function” categories). The number of genes associated with a specific GO term in the gene universe used (“Size”) and number of genes listed as DEGs in cell populations (“Count”) has been indicated. Only GO terms/cell populations with 2 or more genes have been included

	**Cluster**	**GO term**	***p* value**	**Count**	**Size**	**Term**
Biological process	MFC cluster 2	GO:0006412	8.84E − 121	86	403	Translation
	MFC cluster 3	GO:0005975	2.74E − 13	9	355	Carbohydrate metabolic process
	MFC cluster 5	GO:0015669	3.24E − 23	8	28	Gas transport
	MFC cluster 6	GO:0006165	9.35E − 05	2	63	Nucleoside diphosphate phosphorylation
	MFC cluster 7	GO:0008154	8.04E − 08	10	105	Actin polymerization or depolymerization
	RFC cluster 1	GO:0015669	7.90E − 12	4	28	Gas transport
	RFC cluster 2	GO:0006739	4.64E − 08	4	9	NADP metabolic process
	RFC cluster 3	GO:0015669	9.54E − 25	10	28	Gas transport
	RFC cluster 4	GO:0006757	2.97E − 14	6	53	ATP generation from ADP
	RFC cluster 5	GO:0006412	1.32E − 52	51	403	Translation
	RFC cluster 6	GO:0006412	1.02E − 129	104	403	Translation
	RFC cluster 7	GO:0006412	8.01E − 94	92	403	Translation
	RFC cluster 8	GO:0006757	2.52E − 20	10	53	ATP generation from ADP
	RFC cluster 9	GO:0015669	5.58E − 23	10	28	Gas transport
Molecular function	MFC cluster 1	GO:0005509	5.33E − 06	5	937	Calcium ion binding
	MFC cluster 2	GO:0003735	3.85E − 148	88	231	Structural constituent of ribosome
	MFC cluster 3	GO:0004332	9.42E − 06	2	8	Fructose-bisphosphate aldolase activity
	MFC cluster 5	GO:0019825	1.68E − 22	8	31	Oxygen binding
	MFC cluster 6	GO:0003774	6.12E − 14	8	195	Cytoskeletal motor activity
	MFC cluster 7	GO:0016818	3.99E − 08	26	790	Hydrolase activity, acting on acid anhydrides, in phosphorus-containing anhydrides
	RFC cluster 1	GO:0019825	1.29E − 11	4	31	Oxygen binding
	RFC cluster 2	GO:0003779	5.06E − 07	11	377	Actin binding
	RFC cluster 3	GO:0019825	3.22E − 25	10	31	Oxygen binding
	RFC cluster 4	GO:0004332	8.07E − 06	2	8	Fructose-bisphosphate aldolase activity
	RFC cluster 5	GO:0003735	6.27E − 61	47	231	Structural constituent of ribosome
	RFC cluster 6	GO:0003735	9.80E − 169	104	231	Structural constituent of ribosome
	RFC cluster 7	GO:0003735	9.20E − 127	92	231	Structural constituent of ribosome
	RFC cluster 8	GO:0003824	6.68E − 06	21	6943	Catalytic activity
	RFC cluster 9	GO:0019825	1.53E − 22	10	31	Oxygen binding

**Table 3:** Genes defining different cell populations (adjusted *p* value < 0.05) in RFCs/MFCs involved in antigen processing/presentation and HIF-1 signaling

		**1**	**3**	**6**	**7**	**2**	**4**	**5**	**6**	**7**	**8**
Antigen processing and presentation	*cats* (K01368)					0.61					
	*ctsba* (K01363)				0.78					1.12	
	ENSSSAG00000064230 (K01369)				0.95					1.09	
	ENSSSAG00000002454 (K01363)				0.58					1.52	
	*catl1* (K01365)			0.31	1.59					1.69	
HIF-1 signaling pathway	ENSSSAG00000055896 (K00016)				0.35						
	ENSSSAG00000063793 (K00016)							0.60			
	ENSSSAG00000072436 (K01623)				0.34	0.36					
	ENSSSAG00000039415 (K00510)				0.43	0.31			0.30		
	*aldoaa* (K01623)				0.44	0.88					
	*pfkmb* (K00850)							1.01			0.54
	*gapdhs* (K00134)				0.64	0.63			0.34		
	ENSSSAG00000011384 (K00927)							1.02		0.27	0.41
	*eno1a* (K01689)				0.76	0.67			0.30		
	ENSSSAG00000045045 (K01689)			0.30				1.72			0.63
	ENSSSAG00000116144 (K01689)		0.28				0.26	2.31			1.02
	*ldha* (K00016)		0.48				0.27	2.16			1.02
	ENSSSAG00000065456 (K01623)		0.64				0.44	2.20			1.18
	ENSSSAG00000047233 (K01623)		0.40				0.62	2.33			1.27
	*gapdh* (K00134)	0.26	0.41				0.82	2.04			1.68

KEGG ortholog codes have been indicated and columns emphasized (bold = MFC cell populations, italics = RFS cell populations). Numbers show log twofold change when contrasting the expression level of a gene within a specific cell population with the expression level for the whole sections

### Differential gene expression in RFCs versus MFCs

**Fig. 6:**
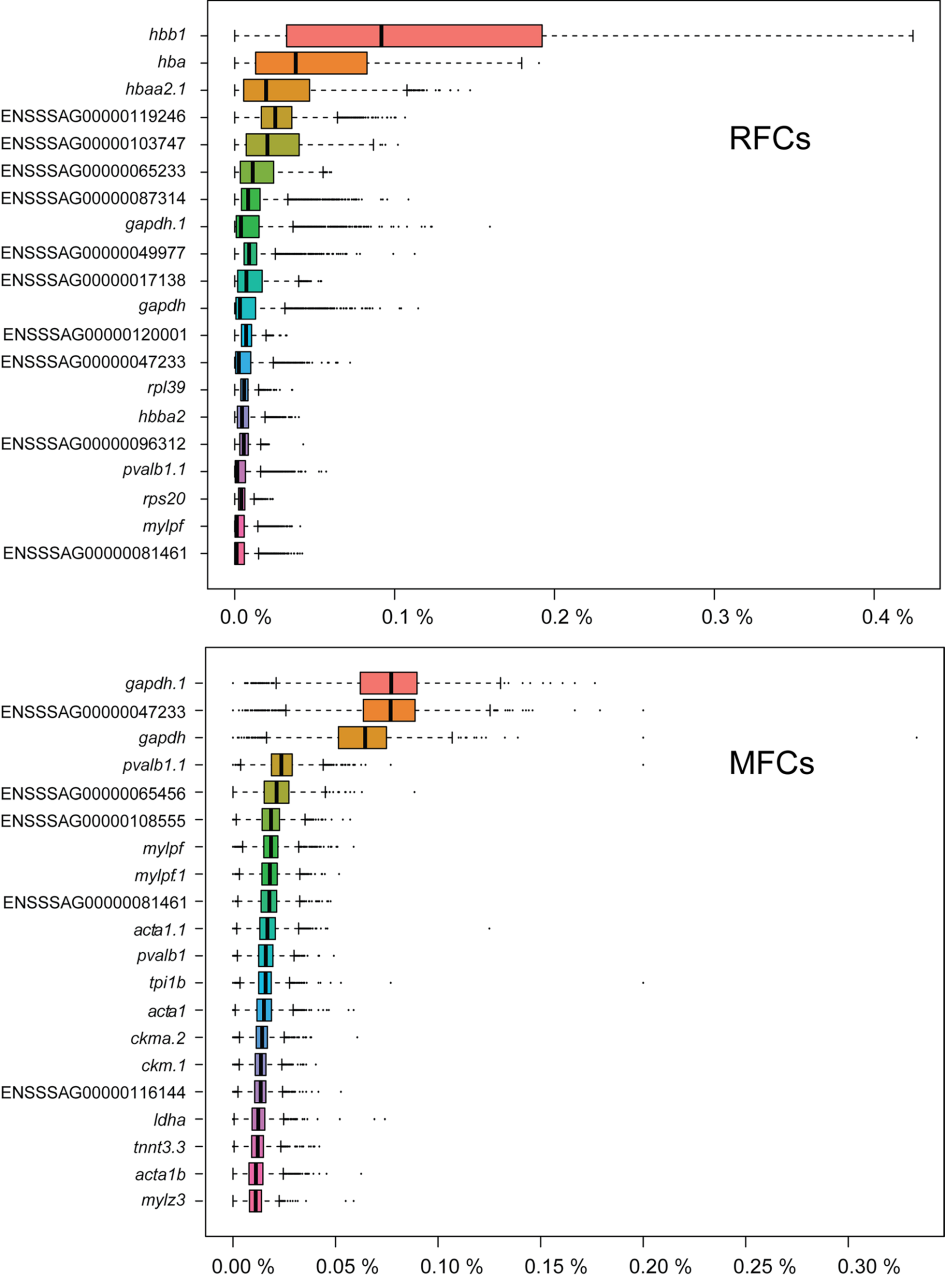
Box plot showing the most highly expressed genes in RFCs and MFCs (percent reads from a specific gene relative to the total number of reads mapped; median values estimated using data from all spots with different spatial IDs)

**Fig. 7:**
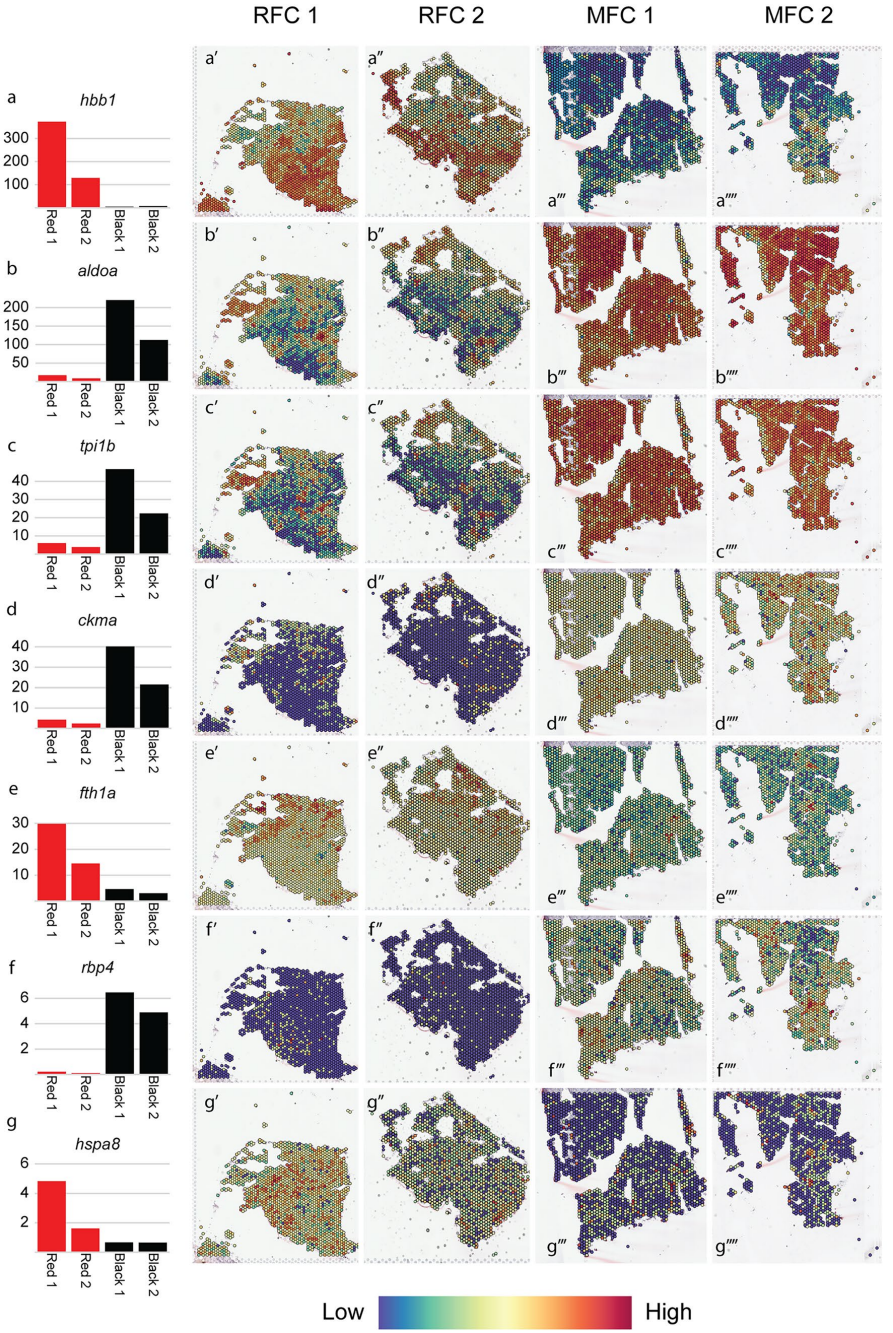
Spatial expression pattern of selected genes (a–g) with corresponding bar diagrams (median-normalized average counts estimated by Space Ranger). Red bars = RFSs and black bars = MFCs. Color scale (bottom) indicates relative expression levels

The most highly expressed genes in RFCs were transcripts associated with erythrocytes and production of hemoglobin, whereas genes involved in energy metabolism and muscle formation/maintenance dominated the list for the MFCs (Fig. 6). Based on transcriptomic profiling of RFCs and MFCs, a number of genes showed a distinct expression pattern between the two groups (Figs. 6 and 7). RFCs showed an upregulation of *hemoglobin subunit beta-1* (*hbb1*). Similarly, a relatively high number of reads were mapped for *ferritin heavy polypeptide 1a* (*fth1a*) in the RFCs, but this gene had a low expression level in the MFCs. *Heat shock protein* (*hspa8*) showed a similar pattern. Contrastingly, *fructose-bisphosphate aldolase A* (*aldoa*) was more pronounced in the MFCs than the RFCs. *Triosephosphate isomerase* (*tpi1b*) transcript levels were also relatively high in the MFCs and *creatine kinase* (*ckma*) and *retinol binding protein* (*rbp4*) had higher expression levels in melanized versus red changes.

### RT-qPCR–PRV-1

For detection of PRV-1 RNA, three out of the four samples (two RFCs and one MFC) were of sufficient quality. The RFCs had Ct values of 25.6 and 27.2, indicating relatively high levels of PRV-1 RNA. In contrast, the MFC sample had a Ct value of 34.

## Discussion

The application of spatial transcriptomics for resolving gene expression profiles has rapidly emerged as a notable and innovative method in molecular biology (Stahl et al. 2016; Marx 2021). The technique provides an unbiased mapping of polyadenylated transcripts, offering the potential for single-cell resolution. By generating a sufficiently high number of mappable reads (100 million +), the resulting data can be equivalent to RNA-Seq libraries with reasonable depth, capturing the transcriptomic profile of thousands of minute biopsies from a small tissue section. In this study, we employed this approach to investigate RFCs and MFCs, a complex and multifactorial condition of unknown etiology.

Representative samples were collected based on visual assessment at the abattoirs, i.e., at the end of the production period of the fish, and histologically evaluated before spatial transcriptomics. In general, RFCs are more homogenous in appearance than MFCs, and are typically characterized by hemorrhage and necrosis, while MFCs display greater heterogeneity (Bjorgen et al. 2019). The MFC samples used in this study corresponds to category 6 changes (Bjorgen et al. 2019), featuring fibrosis and scattered infiltrates of inflammatory cells, including melano-macrophages. However, it should be noted that the sample size used in the current study is significantly smaller than what is commonly used in routine histology, resulting in sections providing limited histological information. Although the essential characteristics were present in our MFCs, these were scattered, with the sections predominantly consisting of unaffected muscle fibers. These served as valuable internal controls in the experiment, contrasting affected and non-affected areas. Nevertheless, in hindsight, the selection could have benefitted from a broader range of samples and sections, ideally containing an abundance of melano-macrophages and granulomatous changes, a hallmark for the most severe MFCs. This highlights the importance of sample collection and the use of standard histological techniques. Even so, the investigated category is representative for a number of observed black spots in the Norwegian salmon production.

The distinct expression patterns of selected genes explains the contrast between the RFCs and MFCs. RFCs are described as hemorrhages, or “bleedings,” within the musculature with aggregates of erythrocytes at the hemorrhage site and necrosis (Bjorgen et al. 2019). An elevated expression level of *hbb1* and *fth1a* was detected in the RFCs which correlates with the presence of erythrocytes (Malik et al. 2021a). RFCs are presumed to correlate with pro-inflammatory microenvironment and could be an earlier initiation point before RFCs progress to become MFCs (Malik et al. 2021a). Upregulation of *hspa8* (alias *hsp70*) supports this argument. Moreover, hspa8 is a potential diagnostic biomarker for the tyrosinase-mediated melanogenesis process and therefore a minor difference of *hspa8* gene expression could be relevant for the transition of RFCs to MFCs (Yin et al. 2021). Melanogenesis affects gene expression associated with glycolysis and enhances the glycolytic activity of enzymes such as aldolase A (Slominski et al. 2014). The relatively high transcriptional level of *aldoa* indicates a higher enzymatic catalytic activity in the MFCs than in the RFCs.

MFCs are characterized by chronic muscle inflammation with necrotic tissue. The creatine kinase gene *ckma* is believed to control cellular energy homeostasis in energy seeking tissues like brain and muscle when compromised/damaged (Baldissera and Baldisserotto 2023). Higher expression of *ckma* in MFCs compared to the RFCs is in agreement with higher muscle degeneration during the chronic inflammatory stage in MFCs.

Our data show that RFCs and MFCs have distinct cell populations that are consistent across biological replicates (Fig. 3). Interestingly, there seems to be a large overlap in the genes defining the different RFCs/MFCs cell populations (Fig. 5) and most of the gene lists are enriched primarily for proteins involved in higher order biological processes (Table 2 and Supplementary Table 1). These observations suggest that many of the same signaling pathways are at play in RFCs and MFCs.

The homogeneity of gene expression pattern within a section is an important factor when the algorithms used define subpopulations of cells, and the number of patterns observed in our data (nine for RFCs and seven for MFCs) might be exaggerated. When defining a cell population, the software makes a compromise where data from multiple genes are combined and reduced to define a specific expression profile. When observing individual genes, only a small fraction seemed to have an expression pattern that convincingly matched a cell population (data not shown). However, the KEGG analysis showed that two signaling pathways of potential relevance for the formation of RFCs and MFCs are markedly affected in several cell populations: antigen processing/presentation and HIF-1 signaling.

Activity in the antigen processing pathway could be attributed to the presence of PRV-1, though specific spatial mapping of PRV-1 transcripts and/or genome fragments was not possible due to the absence of polyadenylation both in genomes and transcripts of reoviruses. It is noteworthy that the viral RNA load was higher in the earlier RFC phase than in the later MCF phase, a pattern observed earlier and correlating with the more pro-inflammatory environment of the RFC (Malik et al. 2021a). However, RFCs can occur prior to detectable viral infection at a population level (Bjørgen et al. 2019), and most likely the viral load detected in a RFC depends on the time of infection. A correlation between PRV-1 and MFCs has been indicated due to the consistent finding of PRV-1 in granulomatous changes and the replication of virus in situ (Bjorgen et al. 2020), raising the hypothesis that PRV-1 is a persistent pathogen causing chronic inflammation. Melano-macrophages within MFCs are also positive when staining for PRV-1 using immunohistochemistry (Bjørgen et al. 2015). This aligns with previous research on melano-macrophages, which has suggested a capacity to retain and present antigens (summarized in Agius and Roberts 2003). However, most of these studies have focused on organized melano-macrophage centers within lymphoid organs, rather than on infiltrating melano-macrophages as part of an inflammatory response. These organized centers have been suggested to be akin to the primitive analogues of the germinal centers found in the lymph nodes of birds and mammals (Ferguson 1976; Ellis 1980). In the context of primitive bony fish, such as the Atlantic salmon, typical melano-macrophage centers do not form. Instead, pigmented cells are dispersed more throughout the kidney and spleen (Agius and Roberts 2003). It seems likely that although these cells are less organized, they still can take active part in immune responses and antigen processing.

One explanation for RFCs is local hypoxic conditions within the musculature, but whether the hypoxia is a primary cause, or secondary due to effects of hemorrhaging, is unknown. In hypoxic musculature, HIF-1 is up-regulated, and HIF-1 orchestrates pivotal cellular adaptative mechanisms in response to hypoxic environments including effects on inflammatory cells (McGettrick and O'Neill 2020). In mammals, this involves the transcriptional activation of over 100 downstream genes, that regulate vital biological processes required for cell survival and function (Masoud and Li 2015). Several pathological conditions may cause hypoxia, including respiratory failure, heart failure, inadequate blood flow due to hemorrhaging or other reasons, dysfunctional/low levels of hemoglobin, or chemically induced hypoxia (Lee et al. 2019). Any of these factors could potentially be linked to the genesis of the hypoxia observed in RFCs.

In conclusion, we have established spatial transcriptomic analysis of musculature from farmed Atlantic salmon and visualized key transcriptomic changes between RFCs and MFCs. Our results agree with previous reports on the condition and add further evidence to the transition from RFCs to MFCs and the in situ production of melanin in MFCs. There appears to be consistent heterogeneity in cell populations across RFCs and MFCs for both general metabolic processes and specific pathways of relevance for tissue discoloration.

### Supplementary Information

Below is the link to the electronic supplementary material.

Supplementary file1 (PNG 54 KB)

Supplementary file2 (PNG 78 KB)

Supplementary file3 (XLSX 72 KB)

Supplementary file4 (XLSX 107 KB)

## Data Availability

All the data relevant for this paper are either included (as supplementary material), or there is a reference in the text as to where they can be accessed.
