# Correction to: Cellular heterogeneity in red and melanized focal muscle changes in farmed Atlantic salmon (*Salmo salar*) visualized by spatial transcriptomics

**DOI:** 10.1007/s00441-023-03856-5

**Published:** 2023-12-30

**Authors:** H. Bjørgen, S. Malik, E. Rimstad, M. Vaadal, I. B. Nyman, E. O. Koppang, T. Tengs

**Affiliations:** 1 Unit of Anatomy, Faculty of Veterinary Medicine, Norwegian University of Life Sciences, 1433 Ås, Norway; 2 Department of Breeding and Genetics, 1433 Nofima, Norway; 3 Unit of Virology, Faculty of Veterinary Medicine, Norwegian University of Life Sciences, 1433 Ås, Norway

**Correction to: Cell and Tissue Research** 10.1007/s00441-023-03850-x

The authors regret that the name of Dr. Bjørgen was captured incorrectly in the original article. His name was captured as H. M. Bjørgen instead of H. Bjørgen.

The original article has been corrected.

