# Supplementary figures and images for: Cellular heterogeneity in red and melanized focal muscle changes in farmed Atlantic salmon (Salmo salar) visualized by spatial transcriptomics
Source: Cell Tissue Res. 2023 Dec 13;395(2):199–210. doi: 10.1007/s00441-023-03850-x (PMC10837230; doi:10.1007/s00441-023-03850-x)

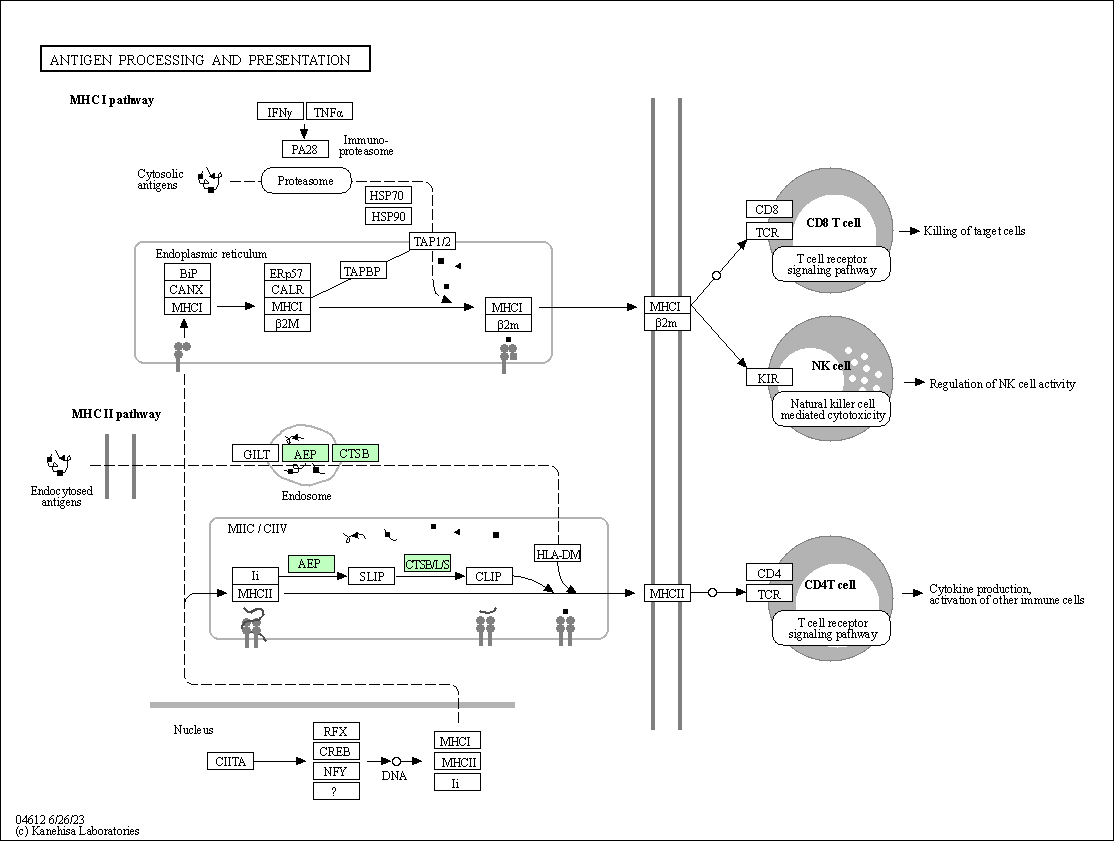

Supplement: Supplementary file 1 — Supplementary file1 (PNG 54 KB) [file 441_2023_3850_MOESM1_ESM.png]

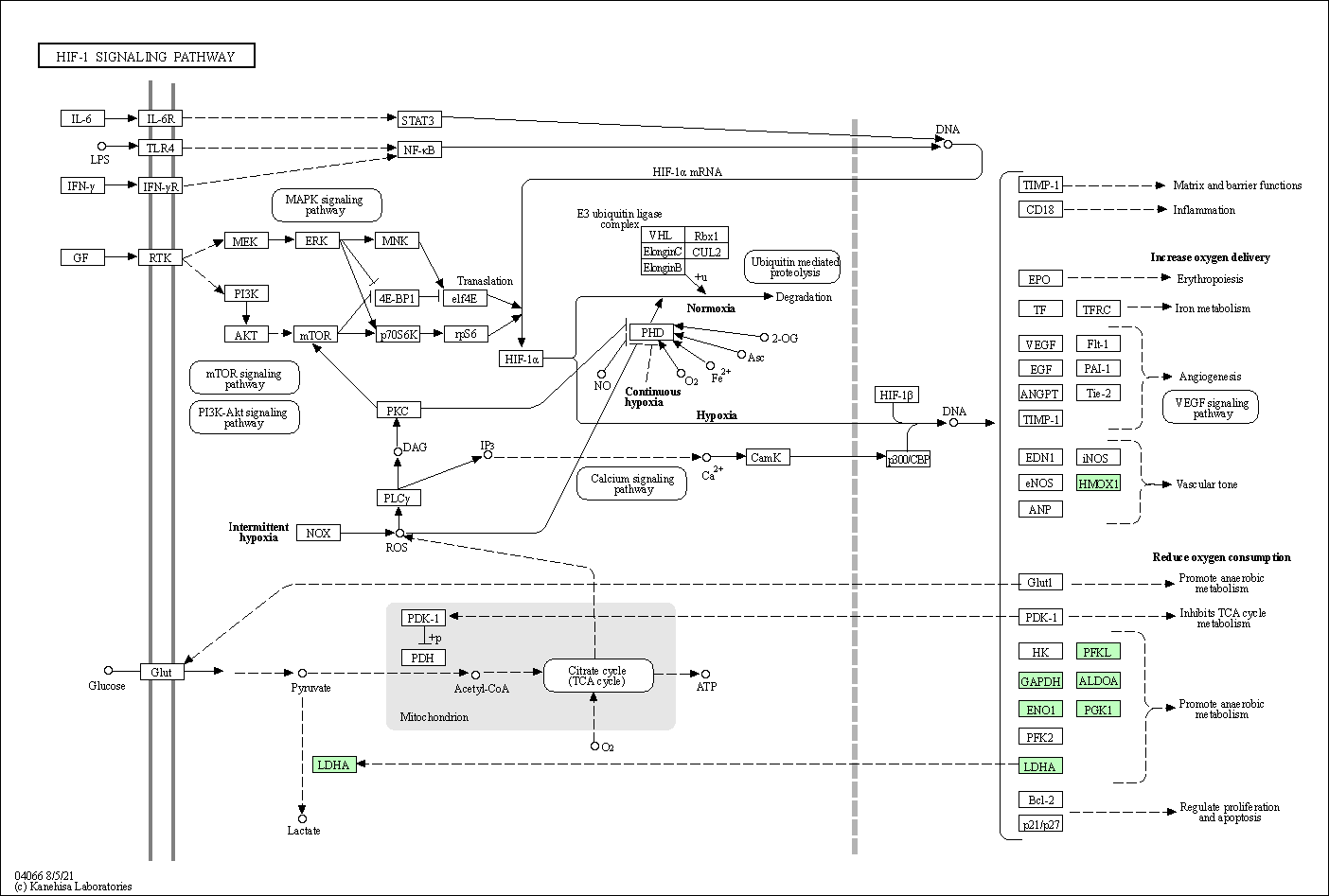

Supplement: Supplementary file 2 — Supplementary file2 (PNG 78 KB) [file 441_2023_3850_MOESM2_ESM.png]
